# miR-144-3p inhibited the growth, metastasis and epithelial-mesenchymal transition of colorectal adenocarcinoma by targeting ZEB1/2

**DOI:** 10.18632/aging.203225

**Published:** 2021-07-05

**Authors:** Taiyuan Li, Cheng Tang, Zhixiang Huang, Lingling Yang, Hua Dai, Bo Tang, Benping Xiao, Jianfeng Li, Xiong Lei

**Affiliations:** 1 Department of General Surgery, The First Affiliated Hospital of Nanchang University, Nanchang 330006, Jiangxi, China; 2 Gastrointestinal Surgical Institute, Nanchang University, Nanchang 330006, Jiangxi, China; 3 Department of Gastroenterology, The Second Affiliated Hospital of Nanchang University, Nanchang 330006, Jiangxi, China; 4 Department of Pathology, The First Affiliated Hospital of Nanchang University, Nanchang 330006, Jiangxi, China; 5 Department of General Surgery, Jiangxi Pingxiang People’s Hospital, Pingxiang 337000, Jiangxi, China

**Keywords:** colorectal cancer, miR-144-3p, epithelial-mesenchymal transition, metastasis, ZEB1/2

## Abstract

miR-144-3p is aberrantly expressed in several types of human cancer and functions as a tumor suppressor by inhibiting metastasis. However, the clinical significance and biological function of miR-144-3p in colorectal adenocarcinoma (CRA) have yet to be elucidated. Here we reported that miR-144-3p expression level was significantly down-regulated in CRA tissues compared with matched noncancerous colorectal mucosae tissues. Low miR-144-3p expression was correlated with adverse clinicopathologic characteristics and poor prognosis of CRA patients. Cox regression analysis showed that low miR-144-3p expression was an independent risk factor for DFS and OS in CRA. *In vitro* and *in vivo* assays showed that miR-144-3p significantly inhibited proliferation, migration and invasion of CRA cells. In particular, miR-144-3p could suppress EMT process of CRA cells by regulating the cytoskeleton and EMT markers. Bioinformatics analysis indicated that EMT associated transcription factors ZEB1 and ZEB2 were potential targets of miR-144-3p, and miR-144-3p inhibited ZEB1 and ZEB2 expression and was negatively correlated with their expression in CRA. Finally, we confirmed that ZEB1 and ZEB2 down-regulation collaboratively mediated the inhibitory effect of miR-144-3p on proliferation, invasion and EMT of CRA cells. In conclusion, our study provided evidence that miR-144-3p could inhibit CRA cell proliferation, invasion and EMT by targeting ZEB1/2.

## INTRODUCTION

Colorectal cancer (CRC) has become the second leading cause of cancer related death, accounting for about 1 in 10 cancer death [1]. About 98% of CRC histopathologic type is colorectal adenocarcinoma (CRA) [2]. Despite improved survival rate of CRA patients due to the development of chemotherapeutic treatments and surgical technology, about 30% CRA patients present with metastasis at the time of diagnosis, which is the major obstacle to improving the prognosis of CRA patients [3]. The liver is the major organ for CRA metastasis, and liver metastasis would develop in about 15% of operable CRA patients and over 50% of inoperable CRA patients [4]. Tumor metastasis involves complex cascade, which is regulated by molecular mechanisms [5]. Therefore, understanding the molecular mechanisms of CRA metastasis cascade will help develop new targeted drugs and improve the survival rate of CRA patients.

Epithelial-mesenchymal transition (EMT) occurs in embryonic developmental stages (e.g. gastrointestinal and neuronal crest formation) and pathological or physiological processes (e.g. wound healing or fibrosis). EMT is often aberrantly activated in tumor cells to gain invasive ability for metastasis [6, 7]. The activation of EMT involves many intracellular signaling pathways that repress E-cadherin expression while up-regulate vimentin expression [8]. EMT associated transcriptional factors, such as SNAI, ZEB and TWIST, are aberrantly upregulated during EMT and participate in CRA metastasis [9]. Accumulating studies demonstrated that miRNAs modulate CRA metastasis via regulating EMT [10, 11].

miRNAs regulate gene expression by preventing translation or promoting mRNA degradation [12]. Alterations in miRNA expression profiles are related to the staging and prognosis of carcinomas [13–15] Recent evidence showed that miR-144-3p may be a tumor suppressor [16–18]. In particular, miR-144-3p inhibited EMT process and cancer metastasis [18–20]. In addition, plasma miR-144-3p level was significantly lower in CRA patients than in healthy controls [21]. However, the significance of miR-144-3p in CRA remains unclear.

In this study, we investigated the expression profile and clinical significance of miR-144-3p in CRA patients and explored molecular mechanisms of miR-144-3p in CRA.

## MATERIALS AND METHODS

### Colorectal adenocarcinoma samples

Fresh frozen colorectal adenocarcinoma tissues (CRAT) and corresponding noncancerous colorectal mucosae tissues (NCMT) harvested from 160 CRA patients at the First Affiliated Hospital of Nanchang University from January 2009 to December 2011 were set as training cohort. In addition, matched NCMTs, CRATs, liver metastatic nodule (LMNs) were obtained from 8 patients with liver-only metastases. Another 140 matched fresh frozen CRATs and NCMTs from CRA patients undergoing radical resection between July 2011 and July 2013 at People's Hospital of Pingxiang were set as validation cohort. None of CRA patients had chemotherapy or radiotherapy before the surgery. This study was approved by Ethics Committee of First Affiliated Hospital of Nanchang University. All participants provided informed consent.

### Follow-up study

Overall survival (OS) indicated the time from the surgery to tumor related death or the last follow-up if the patients survived [2]. Disease-free survival (DFS) indicated the time from the surgery till the patient survived without recurrence or metastasis [2]. All follow-up data were collected for further analysis.

### Cell culture and transfection

Cancer cell lines HCT116, LoVo, SW480, HT-29, SW620 and colorectal mucosal cell line FHC were provided by American Type Culture Collection. Cell were cultured and cell transfection was performed with miR-144-3p mimic, inhibitor and their corresponding control sequences (RiboBio, Guangzhou, China) by using Lipofectamine (Invitrogen, Carlsbad, CA, USA).

### PCR

Total RNA was extracted by using TRIzol (Invitrogen) and cDNA was synthesized by using cDNA kit (Toyobo, Japan). PCR analysis was performed by using SYBR^®^-Green Master kit (Toyobo) (details in Supplementary Materials).

### Western blot analysis

Total proteins were extracted by using RIPA buffer (Beyotime Institute of Biotechnology, Jiangsu, China), and the details for Western blotting were shown in Supplementary Materials.

### Immunohistochemistry (IHC)

The tissue sections were dewaxed, rehydrated, blocked, and then incubated with primary antibodies and horseradish peroxidase conjugated secondary antibody, sequentially (Zhongshan Goldenbridge Biotech, Beijing, China). The sections were then stained with 3,3'-diaminobenzidine substrate.

### Cell proliferation assay

The proliferation ability of CRA cells was analyzed by methyl thiazolyl tetrazolium (MTT), EdU proliferation and colony formation analysis (details in Supplementary Materials).

### Transwell and cell adhesion assays

Cell motility and invasive ability and cell adhesion were examined by transwell migration and invasion assay, cell-cell adhesion and cell-extracellular matrix (ECM) assay, respectively (details in Supplementary Materials).

### Immunofluorescence (IF) and flow cytometry

The details of IF staining and flow cytometry of vimentin and E-cadherin were described in Supplementary Materials. Briefly, cells were fixed and incubated with rhodamine conjugated phalloidin (Solarbio, Beijing, China). The nuclei were stained with DAPI and cells were observed under fluorescence microscope (Nikon Corporation, Japan).

### Animal experiments

CRA cells (5 × 10^6^) were subcutaneously injected into left upper flank of nude male BALB/c mice (4-5 weeks old). Tumor volume was calculated with formula: tumor volume (cm^3^) = (long axis × short axis^2^)/2 [22]. After 6 weeks, tumor tissues were dissected. For *in vivo* metastatic assay, the spleen was exposed after anesthesia and the incision on left lateral flank in nude mice. One month after intrasplenic injection of 1×106 CRA cells, the mice were euthanized and liver specimens were collected to examine metastatic nodules.

### Statistical analysis

All data were analyzed with software SPSS 18 (SPSS, Chicago, IL, USA). The differences between two groups were analyzed by *t* test or χ^2^ test. Survival curve was calculated by Kaplan-Meier method. Factors associated with OS and DFS were identified by Cox proportional hazard regression analysis. *P* < 0.05 indicated significance.

### Ethics approval and consent to participate

The study was approved by the Ethics Committee of the Institutional Review Boards of the First Affiliated Hospital of Nanchang University and Jiangxi Pingxiang People's Hospital, and was performed in accordance with the Declaration of Helsinki and current ethical guidelines. Prior informed consent was obtained from all participants.

### Consent for publication

Patients provided written informed consent for publication. All authors have read and approved of publication of this manuscript.

### Availability of data and materials

The datasets used and/or analyzed during the current study are openly available.

## RESULTS

### miR-144-3p was significantly downregulated in CRA

**Figure 1:**
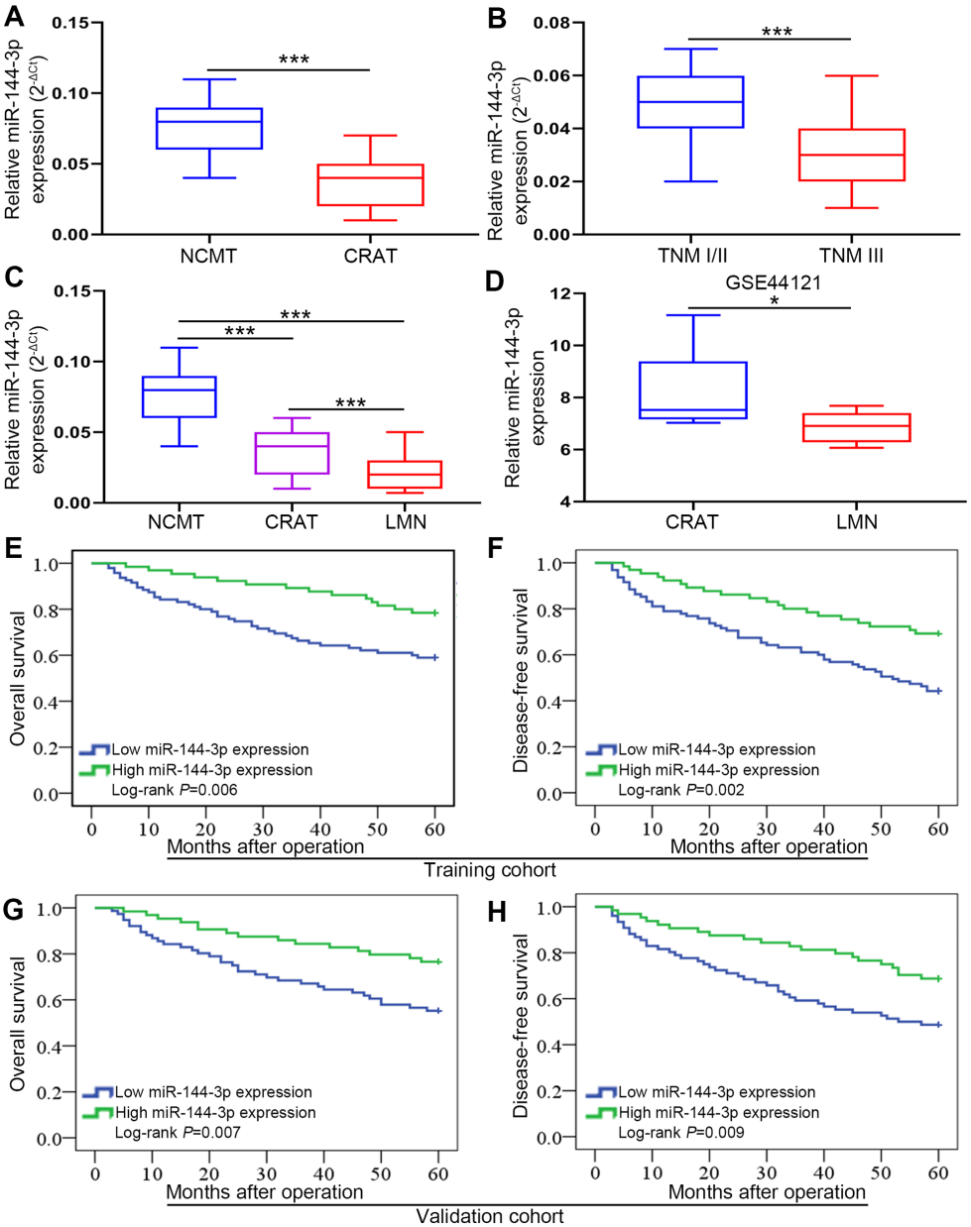
**miR-144-3p expression was significantly downregulated and associated with poor prognosis in CRA.** (**A**) miR-144-3p expression was significantly downregulated in CRATs. The levels of miR-144-3p in 160 paired CRATs and NCMTs from training cohort were determined by qRT-PCR. Data were analyzed using 2^-ΔCt^ method. (**B**) Expression levels of miR-144-3p in CRATs with advanced TNM stage (TNM III) were significantly lower than in early TNM stage (TNM I/II). (**C**) miR-144-3p expression level gradually increased from LMNs, CRATs to NCMTs. The expression levels of miR-144-3p were determined and compared in 8 matched NCMTs, CRATs, and LMNs. (**D**) Data from GSE44121 were used to compare miR-144-3p expression levels in LMNs and CRATs. Overall survival (**E**) and disease-free survival (**F**) were compared by Kaplan-Meier method based on miR-144-3p expression in the training cohort. Overall survival (**G**) and disease-free survival (**H**) were compared by Kaplan-Meier method based on miR-144-3p expression in the validation cohort. NCMTs, noncancerous colorectal mucosae tissues; CRATs, colorectal adenocarcinoma tissues; LMNs, liver metastatic nodules. *, *P*<0.05; ***, *P* < 0.001.

The analysis of 160 pairs of fresh frozen CRATs and corresponding NCMTs from training cohort showed that miR-144-3p level was lower in CRATs than in NCMTs (Figure 1A). Furthermore, miR-144-3p level was lower in CRATs with TNM stage III than in those with TNM stage I/II (Figure 1B). Moreover, analysis of 8 matched NCMTs, CRATs and LMNs demonstrated that miR-144-3p level gradually decreased from NCMTs, CRATs to LMNs (Figure 1C). Analysis of miR-144-3p level in CRATs and LMNs from the GEO dataset (GSE44121) showed that miR-144-3p level was lower in LMNs than in CRATs (Figure 1D).

### Low miR-144-3p expression was associated with poor prognosis of CRA

Next we explored clinical significance of miR-144-3p in CRA following ReMARK guidelines for prognostic tumor biomarkers [23]. We found that miR-144-3p level was significantly lower in CRATs than in NCMTs (data not shown). According to relative miR-144-3p level in CRATs, CRA patients were further divided into low miR-144-3p expression group (log_2_ fold change of CRAT/NCMT ≤ −1) and high miR-144-3p expression group (log_2_ fold change of CRAT/NCMT > −1). First, we found no differences in clinicopathologic characteristics for CRA patients between training cohort and validation cohort (Table 1). In training cohort, miR-144-3p level was related to adverse clinicopathological features such as tumor size (*P*=0.002), pN stage (*P*=0.002), pT stage (*P*=0.003), distant metastasis (*P*=0.004) and lymphatic/microvascular/nerve invasion (*P*=0.001) (Table 2). Similarly, in validation cohort, miR-144-3p level was related to tumor differentiation (*P*=0.011), pT stage (*P*=0.003), pN stage (*P*=0.027), tumor size (*P*=0.001), distant metastasis (*P*=0.018) and lymphatic/microvascular/nerve invasion (*P*<0.001) (Table 3). Based on follow-up data, CRA patients in training cohort with low miR-144-3p level showed shorter OS (*P*=0.006) and shorter DFS (*P*=0.002) compared to patients with high miR-144-3p level (Figure 1E, 1F). CRA patients in validation cohort with low miR-144-3p level showed shorter OS (*P*=0.007) and DFS (*P*=0.009) compared to patients with high miR-144-3p level (Figure 1G, 1H). Furthermore, Cox proportional hazards regression model identified low miR-144-3p level as a risk factor for both OS and DFS (Tables 3, 4).

**Table 1:** Clinicopathologic characteristics of patients with CRA in the training cohort and validation cohort.

**Clinicopathologic parameters**	**Training cohort**	**Validation cohort**	***P***
**Gender**			
Female	62	58	
Male	98	82	0.639
**Age (y)**			
≤60	71	60	
>60	89	80	0.816
**CEA (ng/ml)**			
≤5	68	65	
>5	92	75	0.560
**Tumor differentiation**			
I/II	75	62	
III/IV	85	78	0.728
**Tumor site**			
Colon	58	54	
Rectum	102	86	0.720
**Tumor size**			
≤5 cm	105	93	
>5 cm	55	47	0.903
**pT stage**			
T1/T2	52	51	
T3/T4	108	89	0.542
**pN stage**			
N0	72	66	
N+	88	74	0.729
**Lymphatic/microvascular/nerve invasion**			
Negative	66	55	
Positive	94	85	0.814
**Distant metastasis**			
Negative	76	72	
Positive	84	68	0.563

*, detected during the follow-up period.

**Table 2:** Correlations between expression of miR-144-3p and clinicopathologic parameters of CRA patients in the training and validation cohort.

**Clinicopathologic parameters**	**Training cohort**					**Validation cohort**			
	**n**	**miR-144-3p expression**		***P***		**n**	**miR-144-3p expression**		***P***
		**Low**	**High**				**Low**	**High**	
**Gender**									
Female	62	40	22			58	27	31	
Male	98	55	43	0.324		82	49	33	0.168
**Age (y)**									
≤60	71	46	25			60	31	29	
>60	89	49	40	0.257		80	45	35	0.611
**CEA (ng/ml)**									
≤5	68	35	33			65	31	34	
>5	92	60	32	0.103		75	45	30	0.175
**Tumor differentiation**									
I/II	75	39	36			62	26	36	
III/IV	85	56	29	0.079		78	50	28	**0.011**
**Tumor site**									
Colon	58	31	27			54	34	20	
Rectum	102	64	38	0.315		86	42	44	0.119
**Tumor size**									
≤5 cm	105	53	52			93	41	52	
>5 cm	55	42	13	**0.002**		47	35	12	**0.001**
**pT stage**									
T1/T2	52	22	30			51	19	32	
T3/T4	108	73	35	**0.003**		89	57	32	**0.003**
**pN stage**									
N0	72	33	39			66	29	37	
N^+^	88	62	26	**0.002**		74	47	27	**0.027**
**Lymphatic/microvascular/nerve invasion**									
Negative	66	29	37			55	18	37	
Positive	94	66	28	**0.001**		85	58	27	**<0.001**
**Distant metastasis**									
Negative	76	36	40			72	32	40	
Positive	84	59	25	**0.004**		68	44	24	**0.018**

**Table 3:** Cox proportional hazard regression analysis for OS in the training and validation cohort.

**Parameters**	**Training cohort**					**Validation cohort**			
	**Univariate analysis**	***P***	**Multivariate analysis**	***P***		**Univariate analysis**	***P***	**Multivariate analysis**	***P***
	**HR (95% CI)**		**HR (95% CI)**			**HR (95% CI)**		**HR (95% CI)**	
**Gender** (male *vs* female)	0.819 (0.476–1.409)	0.470		NA		1.183 (0.650-2.153)	0.583		NA
**Age** (y, >60 *vs* ≤60)	1.020 (0.593–1.756)	0.942		NA		1.497 (0.774-2.893)	0.230		NA
**CEA (ng/ml)** (ng/ml, >5 *vs* ≤5)	1.785 (0.993–3.210)	0.053		NA		1.490 (0.823-2.696)	0.188		NA
**Tumor differentiation** (III/IV *vs* I/II)	1.395 (1.081–1.800)	**0.011**	1.432 (0.776–2.643)	0.251		2.467 (1.357-4.485)	**0.003**	1.757 (0.936-3.297)	0.079
**Tumor site** (Colon *vs* Rectum)	1.194 (0.656–2.174)	0.563		NA		1.412 (0.790-2.522)	0.244		NA
**Tumor size** (cm, >5 *vs* ≤5)	1.302 (1.033–1.640)	**0.025**	2.336 (1.229–4.439)	**0.010**		1.934 (1.012-3.699)	**0.046**	1.717 (0.873-3.374)	0.117
**pT stage** (T3/T4 *vs* T1/T2)	1.849 (1.214–2.818)	**0.004**	1.686 (1.213–2.343)	**0.002**		2.317 (1.194-4.498)	**0.013**	2.449 (1.319-4.547)	**0.005**
**pN stage** (N^+^ *vs* N0)	2.172 (1.424–3.314)	**<0.001**	1.901 (1.241–2.913)	**0.003**		2.311 (1.242-4.302)	**0.008**	2.302 (1.180-4.492)	**0.014**
**Lymphatic/microvascular/nerve invasion** (Positive *vs* Negative)	2.144 (1.196–3.844)	**0.010**	2.318 (1.059–5.076)	**0.035**		2.132 (1.188-3.825)	**0.011**	2.367 (1.134-4.938)	**0.022**
**miR-144-3p expression** (High *vs* Low)	0.437 (0.237–0.806)	**0.006**	0.471 (0.254–0.874)	**0.017**		0.443 (0.241-0.814)	**0.007**	0.500 (0.283-0.884)	**0.027**

**Table 4:** Cox proportional hazard regression analysis for DFS in the training and validation cohort.

**Parameters**	**Training cohort**					**Validation cohort**			
	**Univariate analysis**	***P***	**Multivariate analysis**	***P***		**Univariate analysis**	***P***	**Multivariate analysis**	***P***
	**HR (95% CI)**		**HR (95% CI)**			**HR (95% CI)**		**HR (95% CI)**	
**Gender** (male *vs* female)	0.728 (0.459–1.154)	0.177		NA		1.230 (0.712-2.126)	0.459		NA
**Age** (y, >60 *vs* ≤60)	1.138 (0.714–1.814)	0.588		NA		1.661 (0.888-3.107)	0.113		NA
**CEA (ng/ml)** (ng/ml, >5 *vs* ≤5)	1.582 (0.975–2.568)	0.063		NA		1.625 (0.932-2.836)	0.087		NA
**Tumor differentiation** (III/IV *vs* I/II)	1.288 (1.018–1.630)	**0.035**	1.493 (0.844-2.641)	0.169		2.636 (1.484-4.682)	**0.001**	1.693 (0.924-3.104)	0.089
**Tumor site** (Colon *vs* Rectum)	1.097 (0.625–1.924)	0.747		NA		1.089 (0.631-1.880)	0.760		NA
**Tumor size** (cm, >5 *vs* ≤5)	1.304 (1.044–1.629)	**0.019**	1.489 (0.771–2.873)	0.236		1.826 (1.009-3.306)	**0.047**	1.569 (0.849-2.901)	0.151
**pT stage** (T3/T4 *vs* T1/T2)	1.765 (1.187–2.625)	**0.005**	1.593 (1.196–2.121)	**0.001**		1.954 (1.082-3.529)	**0.026**	1.968 (1.064-3.642)	**0.031**
**pN stage** (N^+^ *vs* N0)	2.027 (1.363–3.014)	**<0.001**	2.271 (1.260–4.092)	**0.006**		2.217 (1.222-4.022)	**0.002**	2.638 (1.499-4.643)	**0.001**
**Lymphatic/microvascular/nerve invasion** (Positive *vs* Negative)	2.241 (1.297–3.874)	**0.004**	2.408 (1.169–4.961)	**0.017**		2.303 (1.326-3.999)	**0.003**	2.169 (1.174-4.007)	**0.013**
**miR-144-3p expression** (High *vs* Low)	0.451 (0.269–0.754)	**0.002**	0.467 (0.276–0.789)	**0.004**		0.496 (0.289-0.852)	**0.009**	0.487 (0.265-0.896)	**0.021**

### miR-144-3p inhibited CRA cell proliferation

**Figure 2:**
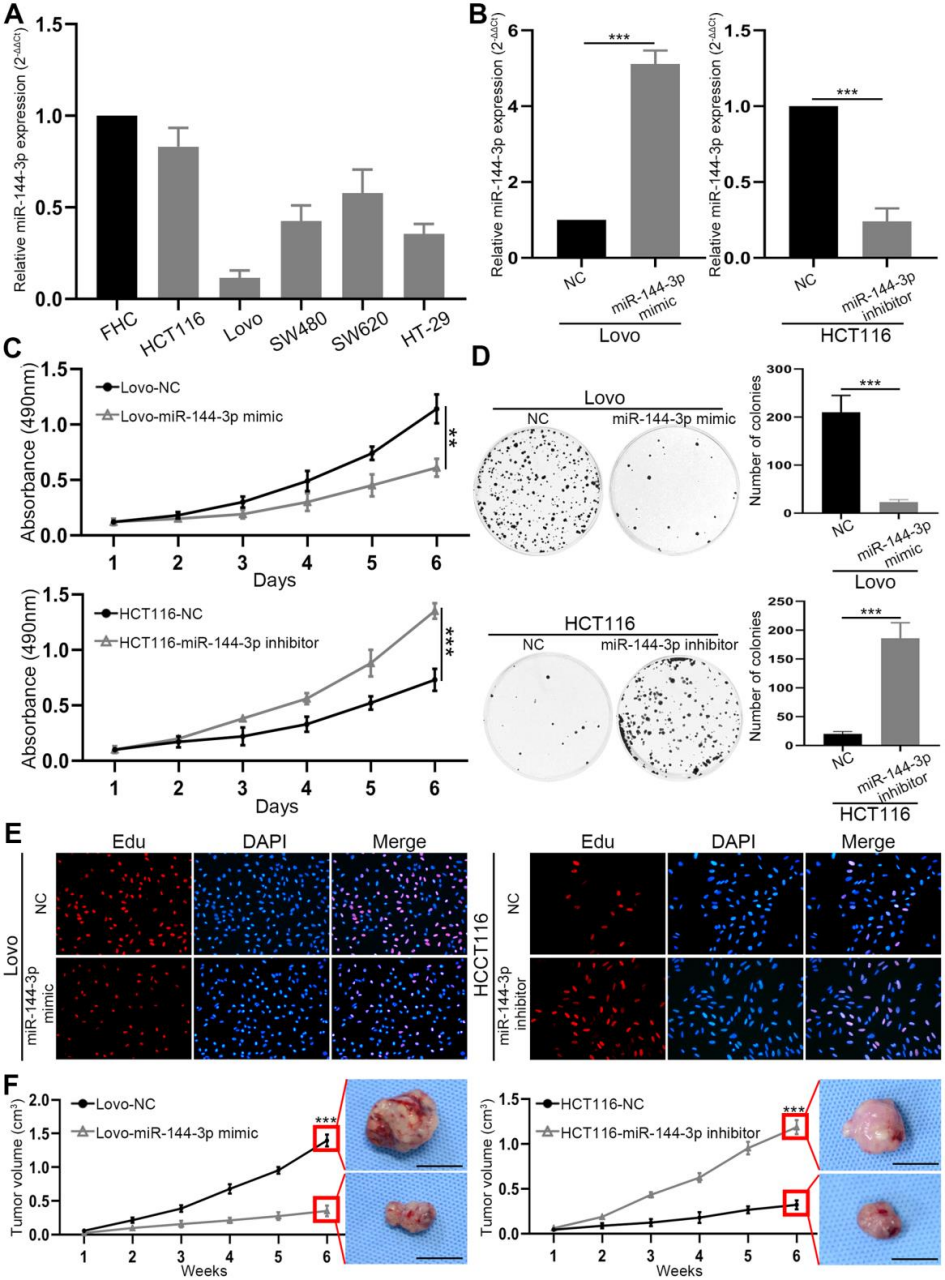
**miR-144-3p was downregulated in CRA cell lines and inhibited CRA cell proliferation and growth.** (**A**) miR-144-3p expression was downregulated in CRA cells lines HCT116, Lovo, SW480, SW620, HT-29 compared to normal colorectal mucosal cell line FHC. (**B**) qRT-PCR was performed to verify the efficiency of overexpression in Lovo cells transfected with miR-144-3p mimic and knockdown in HCT116 cells transfected with miR-144-3p inhibitor. (**C**) MTT assay of Lovo and HCT116 cell proliferation. (**D**) Colony formation assay of Lovo and HCT116 cells. (**E**) Edu assay of the proliferation ability of Lovo cells transfected with miR-144-3p mimic and HCT116 cells transfected with miR-144-3p inhibitor. (**F**) The growth curve showed the change of the volume of subcutaneous tumors from indicated CRA cells and the representative images of subcutaneous tumors harvested 6 weeks after CRA cell inoculation in the right panel. Scale bar: 1cm. NC, normal control. **, *P*<0.01; ***, *P* < 0.001.

Next we explored biological function of miR-144-3p in CRAPCR analysis of miR-144-3p expression in CRA cell lines and colorectal mucosal cell line FHC showed lower expression of miR-144-3p in CRA cell lines than in FHC cell line (Figure 2A). In particular, HCT116 had the highest and Lovo had the lowest level of miR-144-3p, and they were selected for subsequent assays. Transfection of miR-144-3p mimic effectively increased miR-144-3p level in Lovo cells and transfection of mR-144-3p inhibitor reduced miR-144-3p level in HCT116 cells (Figure 2B).

MTT assay showed that miR-144-3p mimic inhibited Lovo cell proliferation, while miR-144-3p inhibitor significantly promoted HCT116 cell proliferation (Figure 2C). Moreover, miR-144-3p mimic significantly inhibited colony formation of Lovo cells, while miR-144-3p inhibitor significantly enhanced colony formation of HCT116 cells (Figure 2D). Edu assay showed that miR-144-3p mimic reduced Lovo cell proliferation and miR-144-3p inhibitor increased HCT116 cell proliferation (Figure 2E). By using subcutaneous xenograft tumor model, we found that tumors derived from Lovo cells treated with miR-144-3p mimic were significantly smaller (Figure 2F). In contrast, miR-144-3p inhibitor promoted the growth of tumors derived from HCT116 cells (Figure 2F).

### miR-144-3p inhibited CRA metastasis

**Figure 3:**
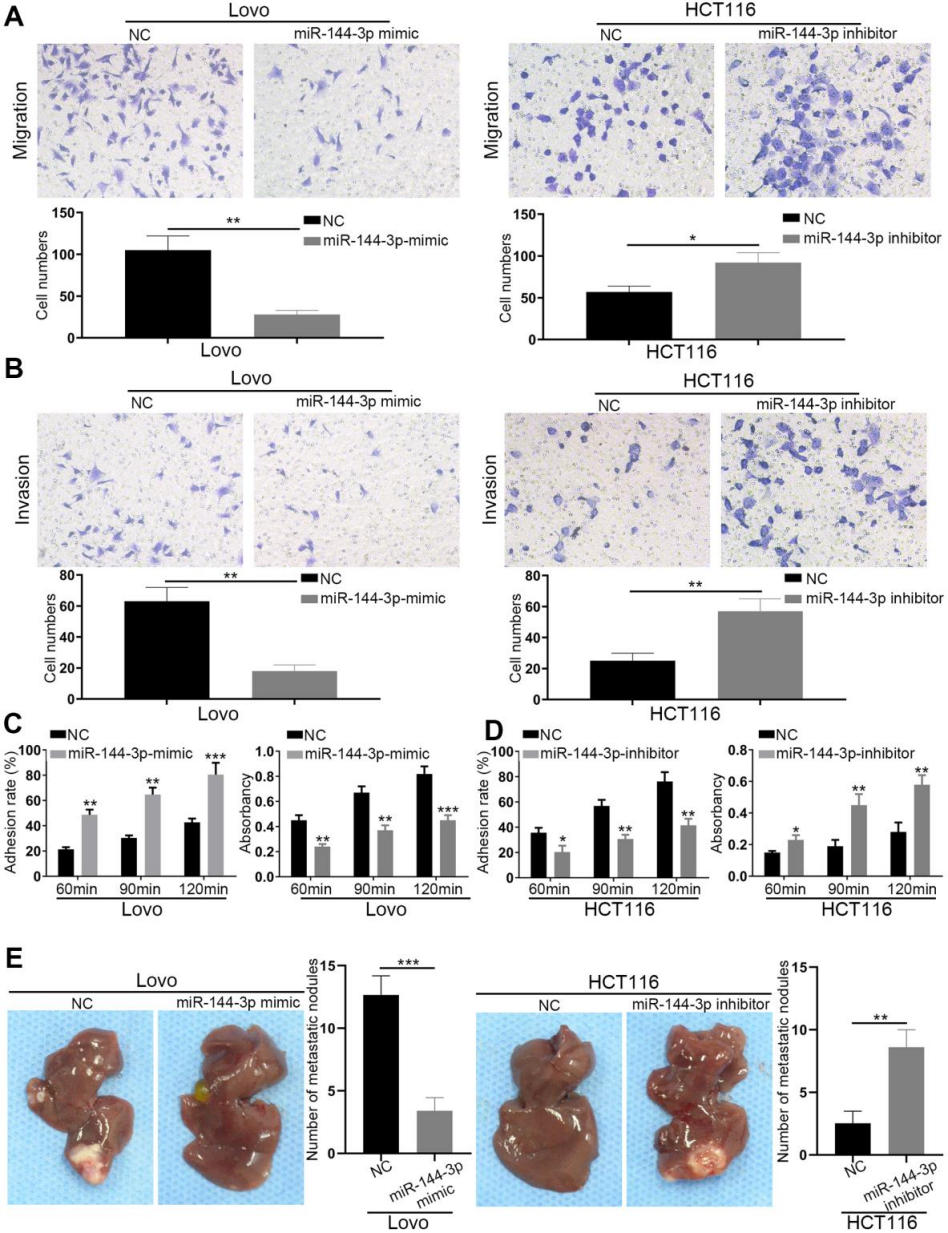
**miR-144-3p inhibited CRA cell migration, invasion and tumor metastasis.** (**A**) Transwell migration assay of migratory ability of Lovo cells transfected with miR-144-3p mimic and HCT116 cells transfected with miR-144-3p inhibitor. (**B**) Transwell invasion assay of invasive ability of Lovo cells transfected with miR-144-3p mimic and HCT116 cells transfected with miR-144-3p inhibitor. (**C**) Overexpression of miR-144-3p in Lovo cells significantly enhanced cell-cell adhesion and decreased cell-ECM adhesion. (**D**) Knockdown of miR-144-3p in HCT116 cells significantly inhibited cell-cell adhesion and increased the cell-ECM adhesion. (**E**) *In vivo* metastatic assays by splenic injection showed that miR-144-3p inhibited CRA liver metastasis. The number of liver metastatic nodules from mice with inoculation of Lovo^miR-144-3p mimic^ cells was significantly smaller than that from mice with inoculation of control cells, whereas the number of liver metastatic nodules from mice with inoculation of HCT116^miR-144-3p inhibitor^ cells was significantly larger than that from mice with inoculation of control cells. ECM, extracellular matrix. *, *P*<0.05; **, *P*<0.01; ***, *P* < 0.001.

Transwell migration assays showed that miR-144-3p mimic significantly inhibited Lovo cell migration, while miR-144-3p downregulation in HCT116 significantly increased cell migration (Figure 3A). Transwell invasion assays showed similar results (Figure 3B). Adhesion assays showed that miR-144-3p mimic significantly enhanced cell-cell adhesion but decreased cell-ECM adhesion in Lovo cells. However, miR-144-3p inhibitor significantly decreased cell-cell adhesion but increased cell-ECM adhesion in HCT116 cells (Figure 3C, 3D). Furthermore, we found lower number of liver metastatic nodules from mice with the injection of Lovo cells transfected with miR-144-3p mimic and higher number of liver metastatic nodules from mice with the injection of HCT116 cells transfected with miR-144-3p inhibitor, compared to control mice (Figure 3E).

### miR-144-3p inhibited EMT

**Figure 4:**
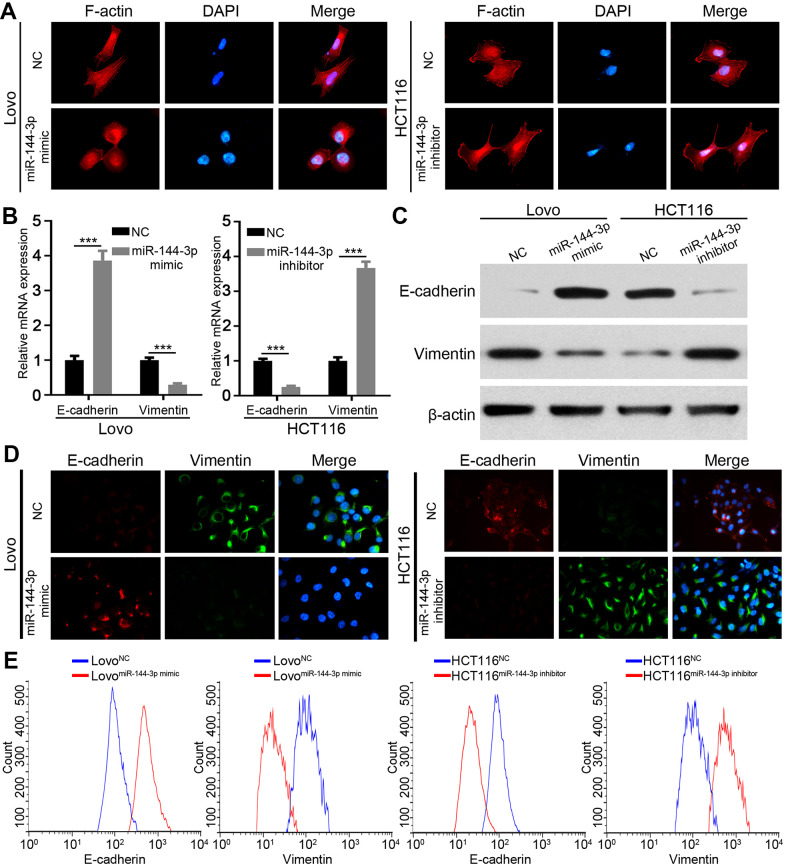
**miR-144-3p inhibited EMT process of CRA cells.** (**A**) Representative images of cytoskeleton showed that miR-144-3p affected polymerization of F-actin and cellular morphology of CRA cells. (**B**) qRT-PCR analysis of the expression level of epithelial marker E-cadherin and mesenchymal marker vimentin in CRA cells. (**C**) Western blot analysis of the expression level of E-cadherin and vimentin in CRA cells. (**D**) Representative IF images showed the expression of E-cadherin and vimentin in CRA cells. (**E**) Flow cytometry analysis of E-cadherin and Vimentin in CRA cells. The results showed that E-cadherin expression was enriched and vimentin expression was reduced in Lovo cells treated with miR-144-3p mimic, while opposite results were observed in HCT116 cells treated with miR-144-3p inhibitor. ***, *P* < 0.001.

Next, we wondered whether miR-144-3p inhibited CRA metastasis by suppressing EMT. We detected actin cytoskeleton of CRA cells based on F-actin staining, because actin transformation is involved in cell adhesion and migration and EMT [24, 25]. Compared to control cells, Lovo cells treated with miR-144-3p mimic showed a cobblestone shape and shrunk F-actin fiber (Figure 4A). In contrast, HCT116 cells treated with miR-144-3p inhibitor presented elongated shape and long F-actin fibers (Figure 4A). Moreover, miR-144-3p mimic promoted E-cadherin expression but inhibited vimentin expression. Inversely, miR-144-3p inhibitor inhibited E-cadherin expression while enhanced vimentin expression (Figure 4B, 4C). Immunofluorescence staining for vimentin and E-cadherin showed similar results (Figure 4D). Flow cytometry showed higher E-cadherin expression and lower vimentin expression in Lovo cells treated with miR-144-3p mimic, while opposite results were shown in HCT116 cells treated with miR-144-3p inhibitor (Figure 4E).

### ZEB1 and ZEB2 are direct targets of miR-144-3p

**Figure 5:**
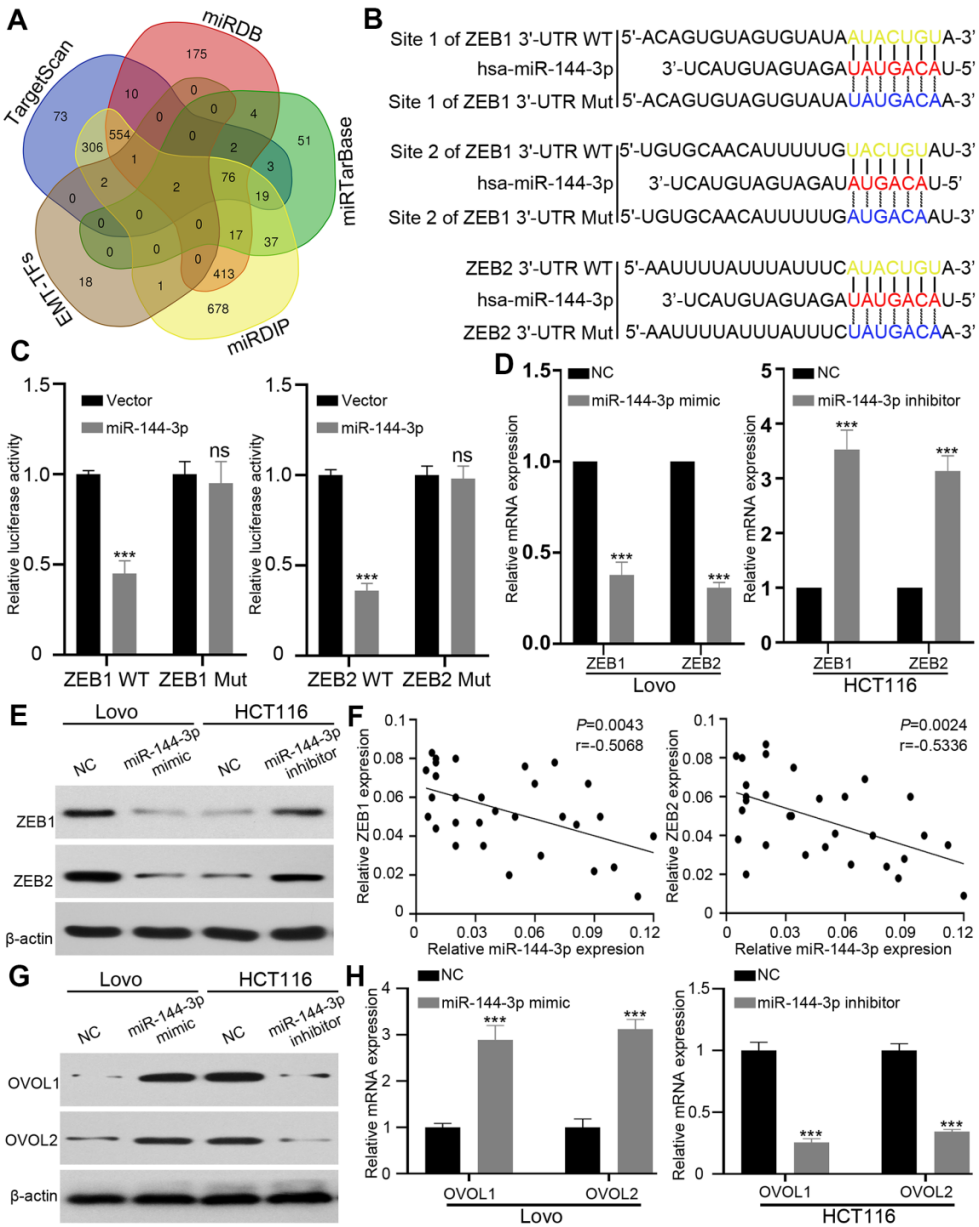
**ZEB1 and ZEB2 were direct targets of miR-144-3p.** (**A**) Venn diagram showed direct targets of miR-144-3p in four public database and crucial EMT-TFs for CRA. Only two potential targets ZEB1 and ZEB2 were presented in all the four database and EMT-TFs. (**B**) The predicted sequences of miR-144-3p binding sites within the 3’-UTR of ZEB1 and ZEB2 including the wild-type (WT) or mutant (Mut) binding site were shown. (**C**) Luciferase reporter assay showed that overexpression of miR-144-3p significantly repressed luciferase activity in 293T cells transfected with WT binding site of ZEB1 and ZEB2 3'-UTR. (D&E) miR-144-3p inhibited ZEB1 and ZEB2 expression. (**D**) qRT-PCR results showed mRNA expression levels of ZEB1 and ZEB2 in Lovo^miR-144-3p mimic^ cells, HCT116 cells^miR-144-3p inhibitor^ and matched control cells. Data were analyzed by using 2^-ΔΔCt^ method. (**E**) Western blot results showed protein expression of ZEB1 and ZEB2 in Lovo^miR-144-3p mimic^ cells, HCT116 cells^miR-144-3p inhibitor^ and matched control cells. (**F**) qRT-PCR analysis of ZEB1 and ZEB2 mRNA expression level in 30 CRATs. Pearson correlation analysis was applied to examine the correlation between miR-144-3p expression and ZEB1 or ZEB2 mRNA expression. (**G**, **H**) miR-144-3p promoted OVOL1/2 expression. (**G**) Western blot analysis of protein expression of transcription factors OVOL1/2. miR-144-3p mimic induced the expression of OVOL1/2 in Lovo cells and miR-144-3p inhibitor reduced OVOL1/2 expression in HCT116 cells. (**H**) miR-144-3p mimic induced mRNA expression of OVOL1/2 in Lovo cells and miR-144-3p inhibitor reduced OVOL1/2 expression in HCT116 cells. TF, transcription factor. ***, *P* < 0.001.

Next we attempted to identify the targets of miR-144-3p, and searched four databases, including TargetScan [26], miRDB [27], miRTarBase [28] and miRDIP, [29]. Among 78 genes predicted by all four databases, we focused on EMT key transcription factors ZEB1, ZEB2 [30, 31]. (Figure 5A). We constructed wild type (WT) luciferase reporter containing 3’-UTR of ZEB1/ZEB2 and mutant type (Mut) luciferase reporter containing mutant binding sequences of ZEB1/ZEB2 for miR-144-3p (Figure 5B). The results of luciferase reporter assay showed that miR-144-3p inhibited luciferase activity of ZEB1 and ZEB2 3’-UTR, but not that of mutant ZEB1 and ZEB2 3’-UTR (Figure 5C). qRT-PCR and Western blot analysis showed that miR-144-3p mimic significantly decreased ZEB1 and ZEB2 expression in Lovo cells while miR-144-3p inhibitor increased ZEB1 and ZEB2 expression in HCT116 cells (Figure 5D, 5E). Furthermore, miR-144-3p level showed negative correlation with ZEB1/2 levels in CRATs (Figure 5F).

ZEB forms a mutually inhibitory feedback loop with transcription factors OVOL1/2 [32]. Therefore, we examined the expression of transcription factors OVOL1/2. While miR-144-3p mimic induced the expression of OVOL1/2 in Lovo cells, miR-144-3p inhibitor suppressed OVOL1/2 expression in HCT116 cells (Figure 5G, 5H).

### ZEB1 and ZEB2 abrogated the inhibition of CRA cell proliferation and invasion by miR-144-3p

**Figure 6:**
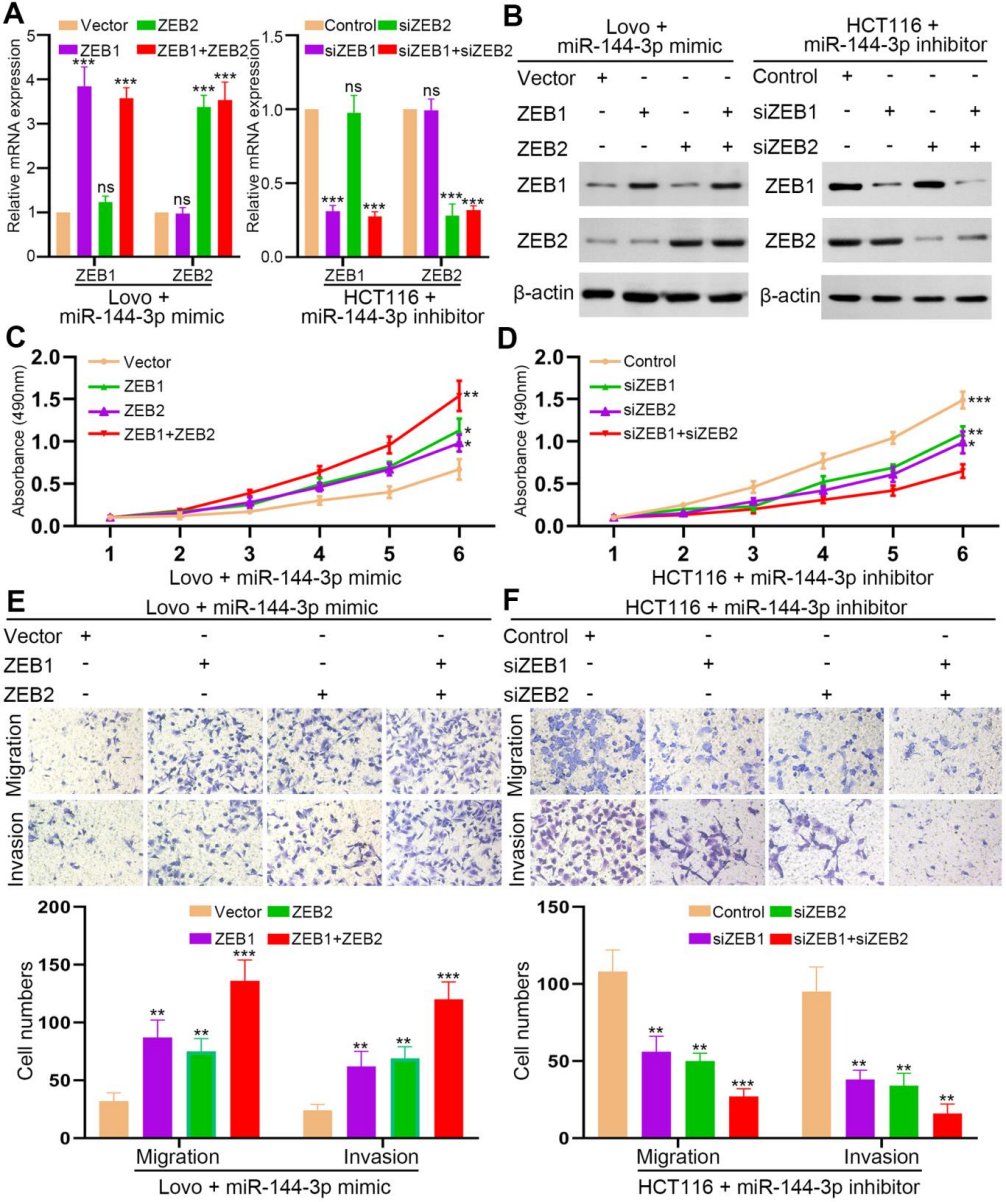
**ZEB1 and ZEB2 collaboratively abrogated the inhibitory effect of miR-144-3p on CRA cell proliferation and invasion.** (**A**) qRT-PCR analysis of ZEB1, ZEB2 expression in Lovo^miR-144-3p mimic^ cells with overexpression of ZEB1 or/and ZEB2 and in HCT116^miR-144-3p inhibitor^ cells with knockdown of ZEB1 or/and ZEB2. (**B**) Western blot analysis of protein expression of ZEB1 and ZEB2 in indicated cells. (**C**) MTT assay of proliferation ability of Lovo^miR-144-3p mimic^ cells. (**D**) MTT assay of proliferation ability of HCT116^miR-144-3p inhibitor^ cells. (**E**) Transwell migration and invasion assays of the migration and invasion of Lovo^miR-144-3p mimic^ cells. (**F**) Transwell migration and invasion assay of HCT116^miR-144-3p inhibitor^ cells after knockdown of ZEB1 or/and ZEB2. *, *P*<0.05; **, *P*<0.01; ***, *P* < 0.001.

Next, we upregulated ZEB1/2 expression in Lovo^miR-144-3p mimic^ cells and downregulated ZEB1/2 expression in HCT116^miR-144-3p inhibitor^ cells (Figure 6A, 6B). MTT assay showed that restoration of ZEB1 or ZEB2 could partly recover the proliferation of Lovo^miR-144-3p mimic^ cells (Figure 6C). However, when both ZEB1 and ZEB2 were elevated, the proliferation of Lovo^miR-144-3p mimic^ cells was completely restored (Figure 6C). In HCT116^miR-144-3p inhibitor^ cells, knockdown of ZEB1 or ZEB2 could partly inhibit enhanced proliferation, which could be fully suppressed by knockdown of both ZEB1 and ZEB2 (Figure 6D). Similarly, the restoration of ZEB1 or ZEB2 alone could partly recover the migration and invasion of Lovo^miR-144-3p mimic^ cells (Figure 6E). The migration and invasion of Lovo^miR-144-3p mimic^ cells were completely restored after the overexpression of both ZEB1 and ZEB2 (Figure 6E). In HCT116^miR-144-3p inhibitor^ cells, knockdown of ZEB1 or ZEB2 could partly inhibit enhanced migration and invasion (Figure 6F), whereas knockdown of both ZEB1 and ZEB2 completely suppressed cell migration and invasion (Figure 6F).

### ZEB1 and ZEB2 mediated the inhibition of EMT by miR-144-3p

**Figure 7:**
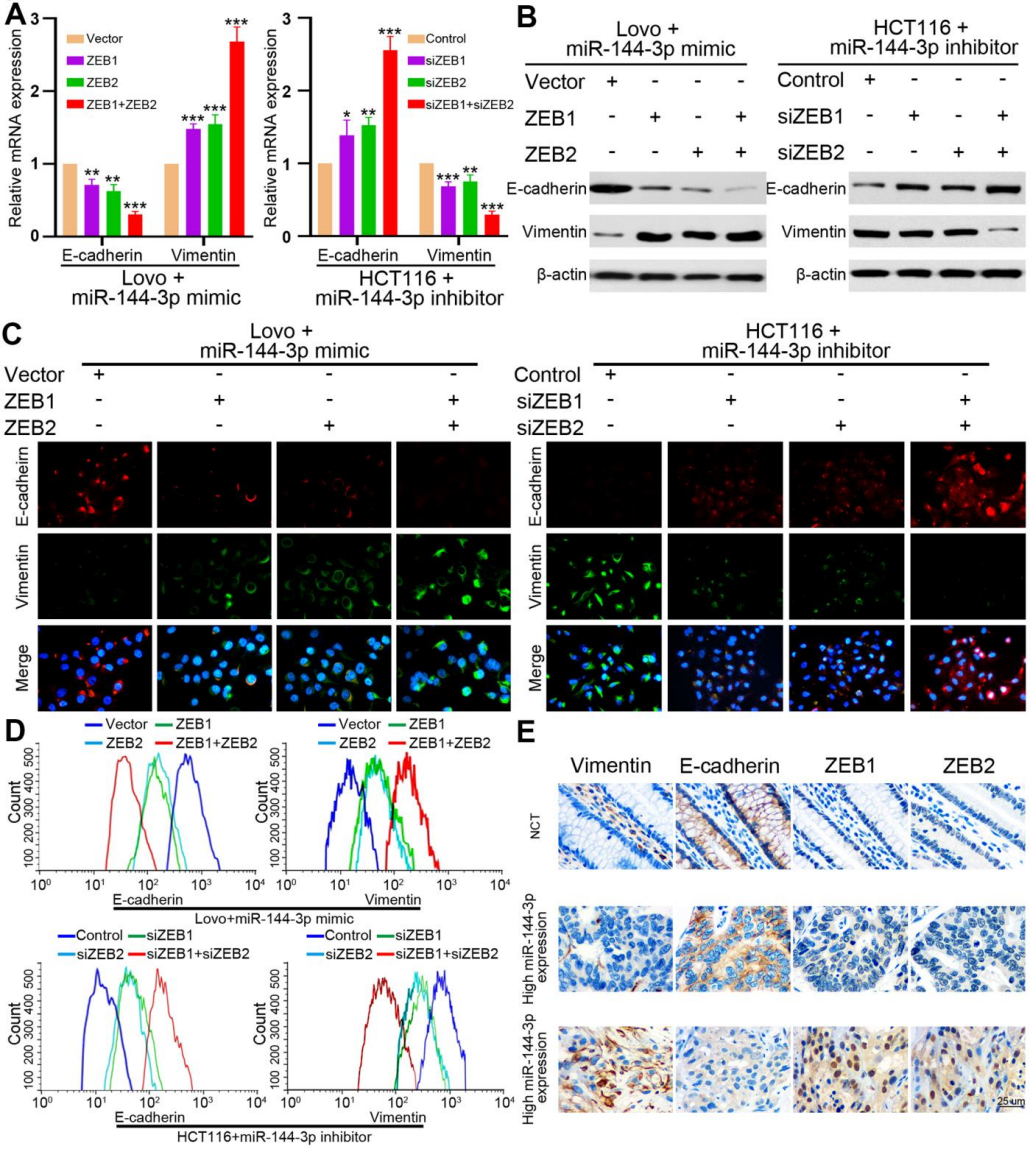
**ZEB1 and ZEB2 collaboratively mediated the effect of miR-144-3p on CRA cell EMT.** (**A**) qRT-PCR analysis of E-cadherin and vimentin expression in Lovo^miR-144-3p mimic^ cells with overexpression of ZEB1 or/and ZEB2, and in HCT116^miR-144-3p inhibitor^ cells with knockdown of ZEB1 or/and ZEB2. (**B**) Protein expression of E-cadherin and vimentin in indicated CRA cells was tested by Western blot analysis. Immunofluorescence (**C**) and flow cytometry (**D**) analysis of E-cadherin and vimentin expression in Lovo^miR-144-3p mimic^ cells with overexpression of ZEB1 or/and ZEB2, and in HCT116^miR-144-3p inhibitor^ cells with knockdown of ZEB1 or/and ZEB2. (**E**) Representative IHC images of serial sections showed the expression of E-cadherin, vimentin, ZEB1, ZEB2 in NCMTs and CRATs with high miR-144-3p expression or low miR-144-3p expression determined by the cut-off value of qRT-PCR. *, *P*<0.05; **, *P*<0.01; ***, *P* < 0.001.

Finally, we examined whether miR-144-3p may inhibit EMT of CRA cells by targeting ZEB1 and ZEB2. In Lovo^miR-144-3p mimic^ cells, the upregulation of ZEB1 or ZEB2 alone could partly upregulate vimentin expression and partly inhibit E-cadherin expression (Figure 7A, 7B). When both ZEB1 and ZEB2 were upregulated, vimentin expression increased while E-cadherin expression decreased significantly (Figure 7A, 7B). In HCT116^miR-144-3p inhibitor^, knockdown of ZEB1 or ZEB2 alone could partly downregulate vimentin expression and upregulate E-cadherin expression, but the effects were better after knockdown of both ZEB1 and ZEB2 (Figure 7A, 7B). Immunofluorescence and flow cytometry confirmed that ZEB1 and ZEB2 mediated the effect of miR-144-3p on EMT in CRA cells (Figure 7C, 7D).

Next we performed IHC to detect E-cadherin, vimentin, ZEB1 and ZEB2 in serial sections of NCMT and CRAT with high or low miR-144-3p expression level, and found that NCMT and CRAT with high miR-144-3p level had low levels of ZEB1, ZEB2 and vimentin and high level of E-cadherin, while CRAT with low miR-144-3p level exhibited opposite levels of these proteins (Figure 7E).

## DISCUSSION

This study showed that miR-144-3p level in CRATs was lower compared to matched NCMTs, and was lower in CRATs with advanced TNM stage than in CRATs with early TNM stage. In addition, miR-144-3p level progressively decreased from matched NCMTs, CRATs to LMNs. These results suggest that miR-144-3p may modulate CRA metastasis.

miRNA expression level has been wildly utilized to assess tumor pathological stages and clinical prognosis [33–35]. In this study, miR-144-3p level in CRA was significantly correlated to aggressive clinicopathological features and poor OS and DFS. In addition, low miR-144-3p level was a risk factor for OS and DFS in CRA. Therefore, miR-144-3p can be a prognostic biomarker for CRA patients after radical surgery.

Studies have shown that miR-144-3p could inhibit tumor proliferation, invasion and metastasis [36–38]. However, its biological function in CRA remains unclear. We demonstrated that miR-144-3p inhibited CRA cell proliferation, invasion and metastasis, indicating its pivotal role in CRA progression. miRNAs play critical role in regulating EMT during CRA development [39]. miR-144-3p could inhibit EMT and metastasis of renal cell carcinoma, gastric cancer, and breast cancer [18–20]. In this study we found that miR-144-3p regulated cell cytoskeleton, decreased vimentin expression while increased E-cadherin expression in CRA cells. These results indicate that miR-144-3p may suppress CRA metastasis by inhibiting EMT process.

ZEB1 and ZEB2 are EMT-associated transcription factors involved in tumor metastasis [40–42]. Based on miRNA target prediction algorithms, both ZEB1 and ZEB2 were predicted to contain binding site of miR-144-3p. We demonstrated that miR-144-3p bound 3'-UTR of ZEB1 and ZEB2, and inhibited their expression in CRA. In addition, ZEB1 and ZEB2 are overexpressed in CRA and are related with poor prognosis for CRA patients [43, 44]. In this study, the levels of miR-144-3p and ZEB1/2 were negatively correlated in CRATs, indicating that miR-144-3p may downregulate ZEB1 and ZEB2 in CRA. We further confirmed that overexpression of ZEB1 or ZEB2 alone could only partly recover inhibitory effects of miR-144-3p on CRA cell proliferation, migration and invasion, while overexpression of both ZEB1 and ZEB2 abrogated inhibitory effects of miR-144-3p on CRA cell proliferation, migration and invasion. Although miR-144 downregulated ZEB1 and ZEB2 in various types of cancers [44, 45], this is the first study to investigate the interaction of ZEB1/2 and miR-144 in CRA. By rescue experiments, we confirmed that ZEB1 and ZEB2 downregulation collaboratively mediated inhibitory effects of miR-144-3p on EMT in CRA. Therefore, our study systematically explored the role of miR-144-3p in CRA.

## CONCLUSIONS

In summary, miR-144-3p was downregulated in CRA and its downregulation was significantly correlated to poor prognosis of CRA patients. Moreover, miR-144-3p inhibited CRA cell proliferation, invasion, and EMT process *in vitro* and suppressed CRA metastasis *in vivo*. Furthermore, we revealed the mechanism that miRNA-144-3p inhibited ZEB1 and ZEB2 expression to suppress CRA growth and metastasis. Therefore, our study suggested that miR-144-3p could be a therapeutic target and prognostic marker for CRA.

## Supplementary Material

Supplementary Materials
