# Supplementary material for: miR-144-3p inhibited the growth, metastasis and epithelial-mesenchymal transition of colorectal adenocarcinoma by targeting ZEB1/2
Source: Aging (Albany NY). 2021 Jul 5;13(13):17349–69. doi: 10.18632/aging.203225 (PMC8312459; doi:10.18632/aging.203225)
Supplement: Supplementary Materials [file aging-13-203225-s001.pdf]

SUPPLEMENTARY MATERIALS

Cell lines

The normal colorectal mucosal cell line FHC, and colorectal adenocarcinoma (CRA) cell line SW480, SW620, HCT116, LoVo, HT-29 cell lines were purchased from the American Type Culture Collection (Manassas, VA, USA). Short tandem repeat (STR) DNA fingerprinting was used to authenticate all cell lines prior to commencement of the study. All cell lines were routinely cultured with RPMI-1640 (Gibco; Thermo Fisher Scientific Inc., Waltham, MA, USA) supplemented with 10% fetal bovine serum (HyClone; GE Healthcare Life Sciences, Logan, UT, USA), and maintained in a 5% CO_2 humidified incubator at 37° C.

qRT-PCR analysis

Total RNA was extracted using TRIzol reagent (Invitrogen; Thermo Fisher Scientific, Inc) according to the manufacturer's protocol. After determining the concentration, the RNA was then reverse transcribed to obtain cDNA using the universal cDNA synthesis kit (Toyobo, Osaka, Japan). qRT-PCR analysis was performed using the SYBR®-Green Realtime PCR Master Mix assay kit (Toyobo, Osaka, Japan). GAPDH and U6 were used as internal controls for gene mRNA and miRNA respectively. The primer sequences were as follows:

miR-144-3p: F: 5'-TACTGCATCAGGAACTGACTG GA-3'; R: 5'-GTGCAGGGTCCGAGGT-3'.

U6: F: 5'-GCTTCGGCAGCACATATACTAAAAT-3'; R: 5'-CGCTTCAGAATTTGCGTGTCAT-3'.

cadherin: F: 5'-CGAGAGCTACACGTTCACGG-3'; R: 5'-GGGTGTCGAGGGAAAAATAGG-3'.

Vimentin: F: 5'-AGTCCACTGAGTACCGGAGAC-3'; R: 5'-CATTTCACGCATCTGGCGTTC-3'.

ZEB1: F: 5'-TTACACCTTTGCATACAGAACCC-3'; R: 5'-TTTACGATTACACCCAGACTGC-3'.

ZEB2: F: 5'-GGAGACGAGTCCAGCTAGTGT-3'; R: 5'-CCACTCCACCCTCCCTTATTTC-3'.

GAPDH: F: 5′-GCACCGTCAAGGCTGAGAAC-3′; R: 5′-TGGTGAAGACGCCAGTGGA-3′.

The results were analyzed using $2^{-\Delta Ct}$ or $2^{-\Delta\Delta Ct}$ method to determine the relative expression level of corresponding genes.

Western blot analysis

Total proteins were extracted by RIPA lysis buffer (Beyotime Institute of Biotechnology, Jiangsu, China). The protein concentrations were measured according to BCA method (Beyotime Institute of Biotechnology). A total of 50 μg protein was subjected to 12% sodium dodecyl sulfate-polyacrylamide gel electrophoresis and then transferred onto PVDF membranes (Millipore; Bedford, MA, USA). Then, the membranes were incubated with primary antibodies and subsequently incubated with the appropriate secondary antibodies (Cell Signaling Technology; Beverly, MA, USA). Bands were detected with enhanced chemiluminescence regents (Thermo Fisher Scientific). β-actin (MilliporeSigma; St Louis, MO, USA) was used as a loading control. The primary antibodies used in this study were as follows: mouse anti-β-actin antibody (MilliporeSigma; St Louis, MO, USA), mouse anti-E-cadherin antibody (CST; Danvers, MA, USA), mouse anti-vimentin antibody (CST), rabbit anti-ZEB1 antibody (Affinity Biosciences, Jiangsu, China), rabbit anti-ZEB2 antibody (Affinity Biosciences).

Cell transfection

miR-144-3p mimic or inhibitor and their corresponding control sequences (RiboBio, Guangzhou, China) were transfected into the indicated CRA cells using Lipofectamine 3000 (Invitrogen; Thermo Fisher Scientific Inc) following the manufacturer's protocol. After 48h transfection, the transfected cells were harvested and verified their efficiency for subsequent experiments. For transfection of ZEB1 and ZEB2 ORF for ZEB1 and ZEB2 overexpression or short hairpin RNA (shRNA) for ZEB1 and ZEB2 knockdown, the lentiviral vectors encoding ZEB1 and ZEB2 ORF or shRNA-ZEB1/2 were synthesized and purchased from GenePharma Co., Ltd (Shanghai, China). The empty vector was used as the negative control. The sequences of shRNAs for ZEB1 and ZEB2 knockdown were as follows: shRNA-ZEB1: 5'-CCUAGUCAGCCACCUUUAATT -3'; shRNA-ZEB2: 5'-GUAAUGACUAGGGCUA UUA-3'. Lentiviral vectors were transfected into cells in 6-well plates using Lipofectamine® 3000 (Invitrogen, Thermo Fisher Scientific Inc.) according the manufacturer's protocol. The efficiency of knockdown or overexpression of ZEB1 and ZEB2 was confirmed by qRT-PCR and western blot. The stably transfected cells were subjected to subsequent experiments.

Cell proliferation and colony formation assays

Methyl thiazolyl tetrazolium (MTT) assays and colony formation assays were used to determine CRA cell proliferation ability. For the MTT assays, 5 × 10^3 cells were seeded into each well of 96-well plates. 100 µl fresh medium containing 0.5 mg/ml MTT (MilliporeSigma) was added into each well and incubated at 37° C for 4 h. The absorbance was measured at 570 nm. For the colony formation assay, about 500 cells were seeded into 35-mm cell-culture dishes (Corning Incorporated; Corning, NY, USA) and cultured for 2 weeks. The number of colonies per dish was calculated following being stained with crystal violet (Beyotime Institute of Biotechnology, Jiangsu, China). Only positive colonies (diameter > 40µm) were calculated and compared. The experiments were repeated three times.

EdU proliferation assays

Cell proliferation was detected using the incorporation of 5-ethynyl-2'-deoxyuridine (EdU) with the EdU Cell Proliferation Assay Kit (Ribobio, Guangzhou, China). Briefly, the indicated cells were seeded in 96-well plates and cultured in complete media under the condition of 5% CO_2, at 37° C. After incubation with 50 µM EdU for 4h, the cells were fixed, permeabilized and stained with EdU in order according to the manufacturer's protocol. Then the cell nuclei were stained with DAPI at a concentration of 1 µg/ml for 30 second. The typical images of the plates were taken using an inverted fluorescence microscope (Nikon Corporation, Tokyo, Japan). The experiments were repeated with three wells.

Transwell assay

Transwell migration and invasion assays were separately used to test CRA cell motility and invasion ability. Briefly, after pre-incubation with 10 µg/ml Mitomycin-C for 1 h to inhibit cell proliferation, about 1x10^5 cells in serum free medium were placed into the upper chamber of the insert. The upper chamber of the insert was coated with Matrigel (BD Biosciences; Franklin Lakes, NJ, USA) used for Transwell invasion assays, while the upper chamber of the insert without Matrigel used for Transwell migration assays. Following incubation for 24 h, the number of cells that adhered to the lower membrane of the inserts was calculated after stained with 0.1% crystal violet (Beyotime Institute of Biotechnology). For each group, the assays were repeated three times, and five random fields of view were selected for analysis.

Adhesion assay

The adhesive ability of the CRA cells was analyzed by cell-extracellular matrix (ECM) and cell-cell adhesion assays. For the cell-ECM adhesion assay, a 96-well plate was plated with fibronectin at 37° C for 60 min and washed twice with DMEM buffer supplied with 0.1% BSA (HyClone, GE Healthcare Life Sciences; Logan, UT, USA). The plates were blocked with DMEM buffer supplied with 0.5% BSA at 37° C in a 5% CO_2 incubator for 60 min. About 1 × 10^4 cells (100 µl cells at a density of about 1x10^5/ml) were added into each well of a 96-well plate (Costar; Corning Incorporated, Corning, NY, USA) and cultured at 37° C. Five wells for each group were determined at 60, 90 or 120 min. After remove of the non-adhesion cells, then 100 µl fresh medium containing 0.5 mg/ml MTT (MilliporeSigma) was added into each well and incubated at 37° C for 4 hours. The wells were then added 100 µl DMSO following remove of the medium and shaken at room temperature for 10 min. Finally, the absorbance was measured at 570 nm. For the cell-cell adhesion assay, sub-confluent (70-80%) cell layers were rinsed twice with Ca_2- and Mg_2-free PBS and separated by incubation in HBSS containing 1 mmol/L EDTA at 37° C for 20 min. And then, 100 µl single cells at density of about 1 × 10^5/ml were added into a 96-well plate (Corning Costar Incorporated) with a fully confluent single cell layer, and cultured at 37° C for 0-120 min. The non-adhesion cells were collected from the wells, and counted using an inverted TE-2000S microscope (Nikon Corporation). The adhesion rate was determined by calculating representative aliquots from each well on a hematocytometer. The adhesion rate was calculated at 60, 90 or 120 min as follow: N0-Nt/N0 ×100%, where Nt is the number of non-adhesion cells at the incubation time t, and N0 is the total number of cells added.

Immunofluorescence (IF)

The CRA cells were grown on the glass coverslips, and then fixed with 4% paraformaldehyde. After permeated in phosphate-buffered saline (PBS) with 0.2% Trion X-100, CRA cells were blocked for an hour with 1% bovine serum albumin (BSA), and then incubated with primary antibody overnight at 4° C. In the following day, the cells incubated with appropriate concentration of secondary antibody (Beyotime Institute of Biotechnology) and DAPI (Beyotime Institute of Biotechnology) separately. Images of the slides were captured using an inverted fluorescence microscope. Primary antibodies for E-cadherin, vimentin were purchased from Santa Cruz Biotechnology (Santa Cruz, CA, USA).

Flow cytometry

CRA cells were seeded in 6-well plates and incubated for 24 h. Then monocytes were washed and incubated with E-cadherin, vimentin antibody (CST; Danvers, MA, USA) at a dilution of 1:10 in phosphate buffered saline (PBS)-bovine serum albumin (BSA) for 30 min at 4° C.

Cells were incubated with an isotype immunoglobulin G (IgG) antibody as control. Cells were filtered through a 70 μM cell strainer immediately prior to flow cytometry, which was carried out on a FACS caliber flow cytometer (BD Biosciences, San Jose, CA).

Luciferase reporter assay

Luciferase activity was assessed according to the Dual-Luciferase Reporter Assay protocol (Promega, Madison, WI, USA) using a Veritas™ 96-well Microplate Luminometer (Promega) with substrate dispenser (Promega). 293T cells infected with miR-144-3p mimic or control were seeded in 96-well plates with 70% confluence. 12 hours later, the cells were co-transfected with 50 ng pGL3-UTR and 10 ng pRLTK by using Lipofectamine LTX. After transfection 24 hours, firefly and Renilla luciferase activities were measured. The Renilla luciferase activities were used to normalize transfection efficiency.
